# Representational Difference Analysis of Transcripts Involved in Jervine Biosynthesis

**DOI:** 10.3390/life10060088

**Published:** 2020-06-19

**Authors:** Magdalena Szeliga, Joanna Ciura, Mirosław Tyrka

**Affiliations:** 1 Department of Biotechnology and Bioinformatics, Faculty of Chemistry, Rzeszow University of Technology, Powstańców Warszawy 6 Ave, 35-959 Rzeszow, Poland; jciura@prz.edu.pl (J.C.); mtyrka@prz.edu.pl (M.T.); 2 Department of Plant Physiology and Biochemistry, Faculty of Biochemistry, Biophysics and Biotechnology, Jagiellonian University, Gronostajowa 7, 30-387 Krakow, Poland

**Keywords:** black false hellebore, cDNA RDA, gene annotation, Next Generation Sequencing (NGS), *Veratrum nigrum* L.

## Abstract

Veratrum-type steroidal alkaloids (VSA) are the major bioactive ingredients that strongly determine the pharmacological activities of *Veratrum nigrum*. Biosynthesis of VSA at the molecular and genetic levels is not well understood. Next-generation sequencing of representational difference analysis (RDA) products after elicitation and precursor feeding was applied to identify candidate genes involved in VSA biosynthesis. A total of 12,048 contigs with a median length of 280 bases were received in three RDA libraries obtained after application of methyl jasmonate, squalene and cholesterol. The comparative analysis of annotated sequences was effective in identifying candidate genes. GABAT2 transaminase and hydroxylases active at C-22, C-26, C-11, and C-16 positions in late stages of jervine biosynthesis were selected. Moreover, genes coding pyrroline-5-carboxylate reductase and enzymes from the short-chain dehydrogenases/reductases family (SDR) associated with the reduction reactions of the VSA biosynthesis process were proposed. The data collected contribute to better understanding of jervine biosynthesis and may accelerate implementation of biotechnological methods of VSA biosynthesis.

## 1. Introduction

Veratrum-type steroidal alkaloids (VSA) are valuable bioactive compounds important for the pharmaceutical industry [1]. Exclusive, natural sources of VSA are limited to the Melanthiaceae family and mainly the *Veratrum* genus [2]. Although, over 40 *Veratrum* species are distributed all over the Northern Hemisphere, only two species (*Veratrum nigrum* L and *V. album*) occur naturally in Europe as a natural source of VSA [3,4,5,6]. *V. nigrum* (2n = 16) belongs to a critically endangered species and a few isolated populations have been reported in Poland [4,7,8]; thus, understanding of VSA biosynthesis is essential for effective biosynthesis of jervine in heterologous systems.

Bioactive benefits of *Veratrum* have been known since ancient times [9]. Pharmacological properties of VSA are used for treatment of dermatitis and contribute to the inhibition of platelet formation in Alzheimer disease [10,11]. Hypotensive, hepatoprotective, analgesic, antimicrobial, anti-inflammatory and antidiabetic activities of VSA have been reported [12,13,14,15,16,17,18,19]. Most importantly, VSA inhibit the Sonic Hedgehog (SHH) signal transduction that is involved in the development of cancer [20]. The inhibition of the activity of the sonic hedgehog pathway may be a strategy to enhance the effects of chemotherapy and may offer new opportunities for cancer treatment [21,22]. Cytotoxic activity of VSA has been found for neuroblastoma [23], glioma [24], melanoma [15], lymphoma [25], promyelocytic leukemia [26], prostate, pancreatic and lung cancer cell lines [27,28,29].

**Figure 1:**
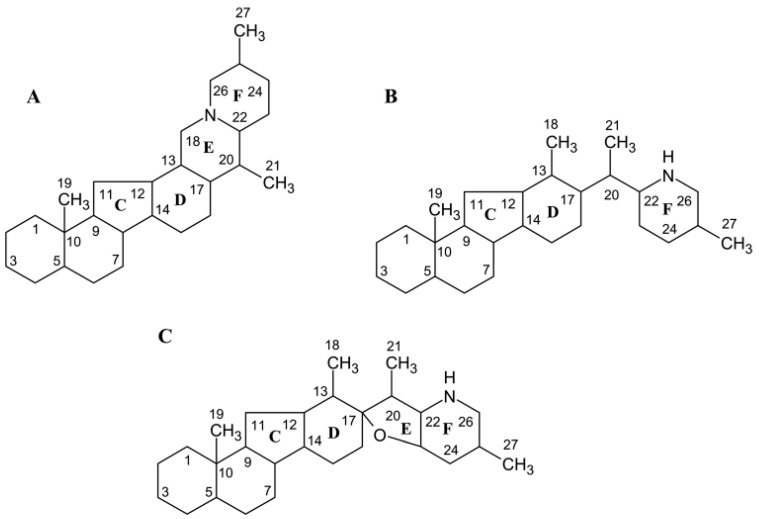
Core structures of three main VSA types: cevanine (**A**); veratramine (**B**); and jervine (**C**).

Diverse VSA can be synthesized by the enzymatic machinery of *Veratrum* spp. and structural aspects allow us to discern cevanine, veratramine and jervine as the main types of VSA. A common feature of VSA is C-nor-D-homo-ring system and the types are characterized by various linkages between the steroidal and alkaloidal components (Figure 1) [15]. There is a demand for low cost production of cyclopamine, a derivative of jervine [30], yet natural resources are limited. Chemical synthesis of VSA is not efficient [31,32]. In *Veratrum* plants, concentration of cyclopamine is generally low (about 0.01%), whereas jervine is more abundant (0.1%) and can be efficiently converted to cyclopamine [33]. The early steps of the jervine biosynthesis pathway leading to cycloartenol and cholesterol have already been described in detail [34,35,36]. Cholesterol is further transformed into solanidine that is the parent compound of VSA; however, corresponding enzymes are generally unknown [30]. Four enzymes that catalyze biosynthesis of cyclopamine [36] have been characterized in *V. californicum*. Recently, organ-specific transcriptomes of *V. nigrum* have been compared to identify 73 candidate genes involved in VSA biosynthesis [37]. 

*Veratrum nigrum* is threatened with extinction and is not cultivated on an industrial scale, so there is a growing interest in application of biotechnological methods to increase VSA production. Genetic transformation of *Veratrum dahuricum* has been developed to increase synthesis of cyclopamine, jervine, and veratramine [38]. Advances in synthetic biology and metabolic engineering also enable the production of plant secondary metabolites in microorganisms. Genome and transcriptome sequencing has become a strategy for discovering genes activated in the biosynthesis of high-value secondary metabolites. These genes can be subsequently used for the gradual reconstruction of the plant metabolic pathway in the cells of microorganisms [39,40,41]. 

Representational difference analysis (RDA) has been developed to identify unique DNA sequences out of two complex and highly related genomes. This method has been adapted for the study of cDNA libraries [42] and for isolating DNA fragments that are differentially methylated [43]. The advantage of the RDA method is its capability of isolating rare mRNAs differentially expressed in two cell populations. RDA can detect sequences represented at 0.0001% in the starting mRNA [44]. The reduction of the complexity is achieved by restriction enzyme digestion, ligation to adaptors and PCR amplification. In the subsequent differential hybridization and amplification steps, sequences in the range of 150–1200 bp unique to the ‘tester’ DNA are enriched [45]. Combination of cDNA RDA with NGS was first used to identify genes involved in diosgenin biosynthesis [46].

Transcriptome sequencing opens the most straightforward way to elucidate biosynthesis of plant specialized metabolites [47]. Substrate feeding and methyl jasmonate signaling to unravel enzymes involved in the biochemical pathway of jervine biosynthesis, with the perspective of designing and optimizing biotechnological process, has been applied. Sequenced libraries enriched with target genes and subtracted background transcripts have been developed in Representational Difference Analysis (RDA).

## 2. Materials and Methods

### 2.1. Plant Material

Seeds of *Veratrum nigrum* (accession 594) were obtained from Masaryk University (Brno, Czech Republic) sterilized and germinated on MS medium [48]. The plants were maintained in vitro at 25 °C with 16/8 h (day/night) photoperiod for one year, and then sprayed (150 µL) with 10% ethanol solutions of cholesterol (CHL) (0.26 mM), methyl jasmonate (MeJ) (0.44 mM) and squalene (SQ) (0’2 mM). Control samples were treated with 10% solution of ethanol. Three plants per treatment were collected after 9 days, frozen immediately in liquid nitrogen and stored at −80 °C until used in phytochemical and transcriptomic analysis. 

### 2.2. Jervine Quantification

Jervine was extracted and quantified by UHPLC-MS/MS as described previously [6]. The one-year-old whole plants of *V. nigrum* from in vitro cultures were ultrasonicated with chloroform and ammonia hydroxide solution (20:3). The residue after evaporation was resolved in methanol and analyzed by ultra-performance liquid chromatography (UHPLC, Shimadzu) coupled with QTRAP 4500 mass spectrometry (AB Sciex) with triple quadrupole. Acetonitrile (solvent A) and water with 0.01% formic acid (solvent B) were used as the mobile phase and separation was carried out using Kinetex XB—C18 LC column (50 mm × 2.1 mm I.D., 1.7 μm). For detecting and measuring jervine content in plant materials, the MRM (multiple reaction monitoring) method was used. The following transitions were analyzed: m/z 425.89/313.10 Da and 425.89/114.0 Da for jervine. Differences in concentration of jervine in fresh weight were analysed by Mann-Whitney U test (with continuity correction) in STATISTICA 12 package (TIBCO Software, Palo Alto, CA, USA). 

### 2.3. Representational Difference Analysis and Library Construction 

RNA was isolated from the whole plants with GeneMATRIX Universal RNA Purification kit (EURx, Gdańsk, Poland). The total RNA was quantified and qualified on Agilent 2100 BioAnalyzer using RNA 6000 Nano Kit (Agilent Technologies, Santa Clara, CA, USA). High quality samples were selected for ProtoScript II First Strand cDNA Synthesis Kit and mRNA Second Strand Synthesis Module (New England Biolabs, Ipswich, MA, USA) to obtain the first and the second strand of cDNA, respectively. Representational difference analysis was performed on cDNA [42] with small modifications. Bulked cDNAs from three plants characterized with the highest jervine contents, obtained after cholesterol, methyl jasmonate and squalene treatment, were used as factor specific testers. Untreated, control plants with the lowest concentration of jervine were bulked and used as a driver. Three rounds of subtractive hybridization were utilized to obtain respective differential libraries of sizes from 200 to 350 bp for subsequent sequencing. The paired-end cDNA sequencing libraries were prepared starting from 1 ng of cDNA per sample using Nextera XT DNA Sample Preparation Kit (Illumina, San Diego, CA, USA) according to manufacturer instructions. The libraries were run (2 × 300 cycles) on the MiSeq (Illumina, San Diego, CA, USA) platform. RNA-sequencing raw data for CHL, MeJ and SQ have been deposited in NCBI SRA database (accessions SRR10256726–SRR10256728). 

### 2.4. De Novo Assembly and Functional Annotation

Raw reads were cleaned by removal of read-through adapter sequences, low quality sequences and ambiguous nucleotides. Clean reads were de novo assembled (Trinity) in CLC Genomics Workbench 12 (Qiagen, Germantown, MD, USA) at default parameters. Cd-hit-est program was used to reduce the redundant unigenes with cut-off set to 0.9 [49]. Unigenes were annotated against non-redundant proteins (NR), nucleotide (NT), EuKaryotic Orthologous Groups (KOG), and Pfam databases using WebMGA server [50] or CLC Genomics Workbench 12. Kyoto Encyclopedia of Genes and Genomes (KEGG) database and Automatic Annotation Server (KAAS) with bi-directional best hit method associated sequences to the KEGG pathways and orthology (KO) assignment [51]. The gene ontology (GO) annotations were analyzed using the Blast2GO program [52] and visualized with WEGO software [53]. The transcription factor (TF) families were identified in the Plant Transcription Factor Database [54]. The expression levels of unigenes were defined by the FPKM values (fragments per kb per million reads) using CLC Genomics Workbench 12 program. Functionally grouped unigenes in RDA libraries were compared (blastn, blastx) with local databases of reference sequences and respective unigenes obtained from organ-specific libraries of *Veratrum nigrum* [37] at the E-value threshold set to 0.05. The contigs in RDA pools were further assembled in Geneious v 8.1.9 in pairwise combinations. Contigs that were not reassembled between two compared pools were treated as unique and specific to RDA library. Sets of contigs were compared at different levels to obtain data for the Venn charts.

## 3. Results and Discussion

### 3.1. Jervine Concentration

**Figure 2:**
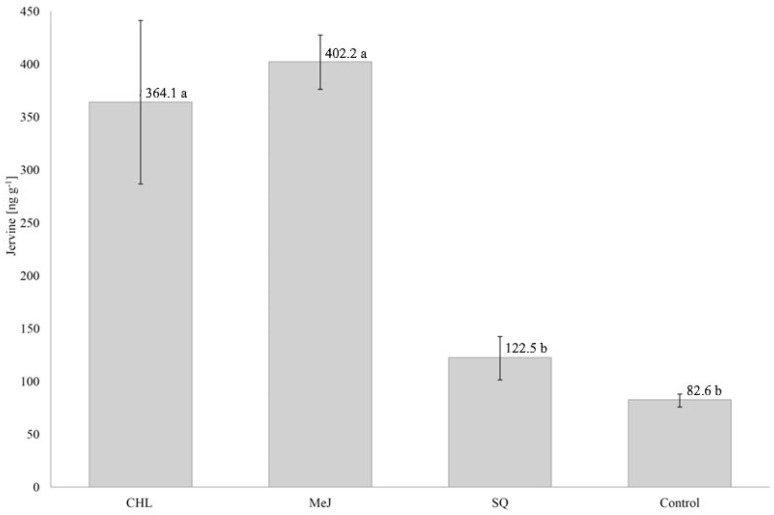
Average jervine content in *V. nigrum* plants after cholesterol (CHL), methyl jasmonate (MeJ) and squalene (SQ) treatment compared to control. Error bars indicate standard deviation (±SD) in three biological replicates. The values sharing the same letter were not significantly different at *p* < 0.05.

*Veratrum nigrum* plants were fed with squalene and cholesterol as precursors in order to overexpress genes involved in jervine biosynthesis. Alternatively, production of secondary metabolites was enhanced by the treatment with methyl jasmonate as elicitor. Average jervine concentration in CHL- and MeJ-treated plants was over four-fold higher than in control plants (Figure 2). The content of jervine in individual plants sprayed with cholesterol varied up to 543.62 ng g^−1^. Jervine biosynthesis was not significantly affected by squalene treatment. The results provide evidence that cholesterol and methyl jasmonate efficiently stimulate jervine accumulation in *Veratrum* plants. Methyl jasmonate has been reported to elicit the production of secondary metabolites in plants, including steroidal alkaloids in *Solanum*. Exposition of transgenic tomato to 100 μM MeJ vapor for 4 days resulted in two-fold increase of α-tomatine level relative to control [55]. Furthermore, the positive effect of cholesterol feeding on the synthesis of steroidal alkaloids was also found in *S. lyratum* cells. Application of cholesterol showed a direct dose effect (0.05, 0.5, and 20 mg L^−1^) on both solanidine and solasodine synthesis [56].

### 3.2. Sequence De Novo Assembly

cDNA libraries prepared after three rounds of subtraction were sequenced in the Illumina technology. A total of 1966, 1849 and 3047 thousand raw reads corresponding to 203, 207 and 342 million bp were generated for CHL-RDA, MeJ-RDA and SQ-RDA libraries, respectively. Cleaning and filtering stages attributed to the selection of 1905, 1810 and 2981 thousand reads for CHL, MeJ and SQ treatments, respectively. A total of 12,048 contigs with a median length of 280 bases were obtained by de novo assembly of clean reads (Table 1). 

**Figure 3:**
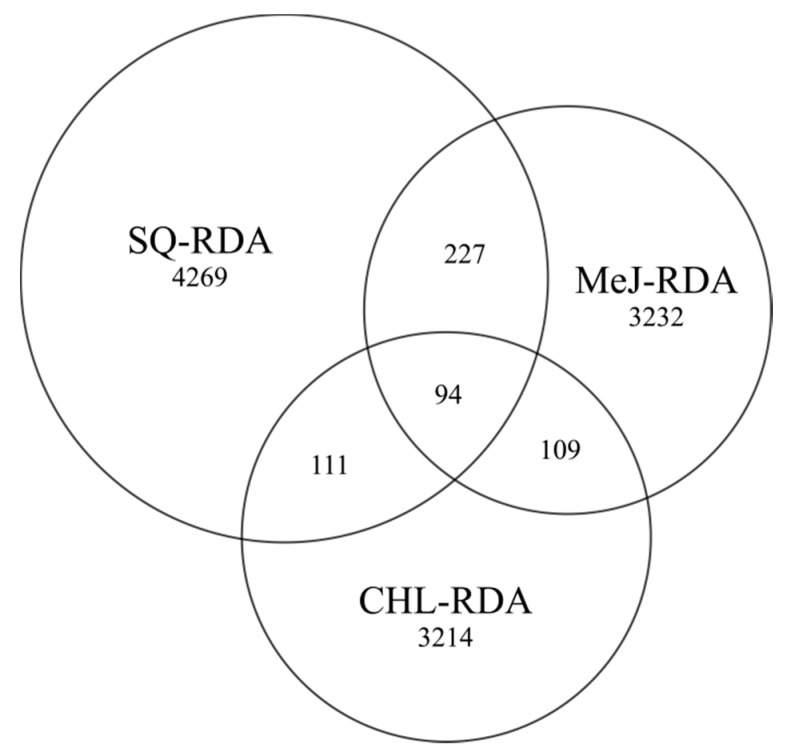
Venn diagram with distribution of unique and common contigs in the SQ-RDA, MeJ-RDA and CHL-RDA subtractive libraries developed after squalene, methyl jasmonate and cholesterol treatment, respectively.

The number of unique transcripts was similar in CHL and MeJ libraries and accounted for 3214 and 3232 unigenes, respectively. The largest amount of unigenes (40%) was obtained for subtracted library after squalene treatment, and 94 (0.8%) contigs were common for the three differential products. Furthermore, the samples after squalene and methyl jasmonate treatment had the highest number of common transcripts (321) compared to other pairs (Figure 3). 

Clustering of transcripts to remove redundancy is a very sensitive step of sequence de novo assembly. Nucleotide identity between allelic variants of CYP genes from Veratrum californicum [36] was above 99%, whereas identity of paralogous genes CYP94N1 vs. CYP94N2, CYP90G1 vs. CYP90G2 and GABAT1 vs. GABAT2 was 95%, 97.5%, and 78.5%, respectively. The presence of extensive duplications in cytochrome P450s and transporter families provide additional reason for an increase of similarity threshold at clustering sage. In this view, the default level of 90% similarity seems to be overly relaxed and may lead to elimination of paralogues and allelic variants. In case of cDNA RDA approach, redundancy reduction is mostly addressed during library preparation and it can be assumed that at least a fraction of highly homologous DNA fragments derived from paralogs have been deleted. Both reduced threshold level at clustering stage and properties of the RDA library preparation method may contribute to underestimating of gene family diversity.

### 3.3. Functional Annotation

The contigs were reduced to 11,891 unigenes and annotated at NT, NR, KOG, Pfam, KEGG and GO databases (Table 2). From 95% to 85% of unigenes representing the three RDA products showed significant similarity to sequences in NT and NR databases. Furthermore, a total of 5562 unigenes showed similarity to proteins in the KOG database. The distribution of the transcripts in 25 KOG categories was similar for all three cDNA-RDA products. The highest number of unigenes in CHL, MeJ and SQ subtractive libraries belonged to the cluster of the posttranslational modification, protein turnover, and chaperones (259, 261 and 313, respectively). 210 unique transcripts were assigned to the cluster of secondary metabolites biosynthesis, transport and catabolism including 16, 24 and 21 unigenes corresponding to cytochrome P450 family in respective libraries. 

Furthermore, a total of 6042 unique transcripts were classified in the Pfam database into 2011 protein domains/families. Among all of the annotated sequences, the most abundant in CHL, MeJ and SQ differential products were protein kinase domain (Pkinase, PF00069.25 with 33, 35, and 46 transcripts, respectively), RNA recognition motif (RRM_1, PF00076.22) and WD domain (WD40, PF00400.32). Cytochrome P450 group (P450, PF00067.22) was represented by the 15, 19 and 17 transcripts from CHL, MeJ and SQ libraries, respectively.

**Figure 4:**
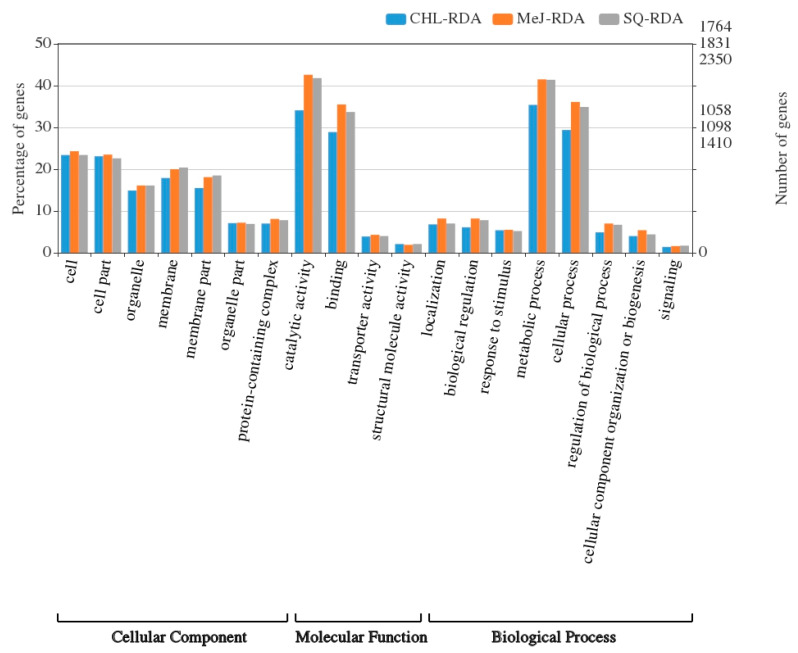
GO classification of unigenes in CHL-RDA, MeJ-RDA and SQ-RDA subtractive libraries.

Unigenes of *V. nigrum* were most frequently (30%) similar to sequences of *Medicago truncatula*. Gene ontology (GO) revealed annotations for 60%, 72% and 70% of unique transcripts in CHL-RDA, MeJ-RDA and SQ-RDA libraries. These sequences were categorized into biological processes, cellular components, and molecular function (35%, 25% and 40%, respectively). Within the above categories, the most abundant subcategories corresponded to metabolic process (GO: 0008152), cell (GO: 0005623) and catalytic activity (GO: 0003824) (Figure 4). Moreover, detailed analysis of unigenes in the metabolic process GO subcategory, revealed sequences (595, 710 and 884 from CHL-RDA, MeJ-RDA and SQ-RDA libraries) assigned to organonitrogen compound metabolic process that includes biosynthesis of steroidal alkaloids. 

**Figure 5:**
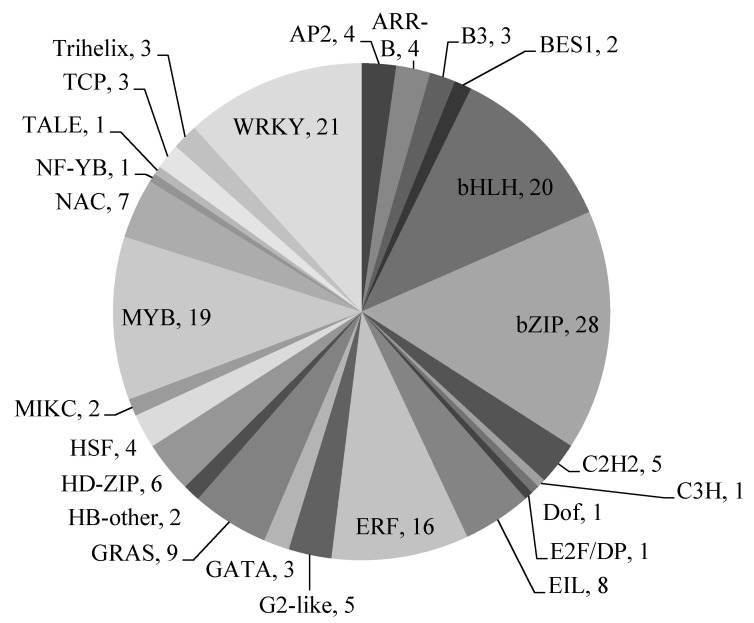
Distribution of *V. nigrum* unigenes in 26 transcription factor families. The total number of sequences are provided by transcription factor (TF) family name.

In the molecular function GO category, a total of 225 unigenes coded transcription factors (TF, GO term: 0003700; DNA-binding transcription factor activity). Most of these unigenes (179) were further separated into 26 TF families at Plant Transcription Factor Database [54]. The most frequently represented TF belonged to basic leucine-zipper (bZIP) superfamily (16%), WRKY family (12%), basic helix-loop-helix (bHLH) (11%), MYB proteins (11%) and ERF superfamilies (9%). (Figure 5). All these families were reported to play an important role in the biosynthesis of sterol backbone, terpenoids and alkaloids. The WRKY transcription factors regulate biosynthesis of alkaloids (e.g., terpene indole alkaloids) and terpenoids (for example sesquiterpenes and diterpenes) [57,58,59,60]. Similar activities were observed for MYC2 and bHLH family proteins [61,62]. Furthermore, the bHLH transcription factors TSAR1 and TSAR2 regulate saponin biosynthesis in *Medicago truncatula* [63]. The MYB transcription factors regulate the flux in various branches of terpene metabolism. For example, the sweet basil data showed that *MsMYB* can affect both monoterpene and sesquiterpene synthesis [64]. Finally, the AP2/ERF transcription factors regulate the biosynthesis of steroidal alkaloids in the Solanaceae family. The *GAME9* from ERF superfamily controls several upstream mevalonate and cholesterol precursor pathway genes in potato and tomato [65]. Identification of transcription factors effective in stimulation of selected biosynthetic pathways facilitates overexpression of these TFs to increase concentration of target compound [66]. 

The unigenes from subtractive libraries were annotated at KEGG database to identify the active biological pathways. In total, unique KO identifiers were allocated to 1415, 1438 and 1845 transcripts from CHL-RDA, MeJ-RDA and SQ-RDA, respectively. All the unique transcripts mapped to 277 predicted metabolic KEGG pathways within five main categories of metabolism (43%), genetic information processing (13%), environmental information processing (13%), cellular processes (12%) and organismal systems (19%) (Table S1). Among these groups, signal transduction, carbohydrate metabolism and amino acid metabolism pathways were most frequently represented (by 1001, 953 and 554 unigenes, respectively). Moreover, 21, 11 and 23 enzymes involved in steroid and terpenoid backbone biosynthesis were identified in CHL-RDA, MeJ-RDA and SQ-RDA libraries.

**Figure 6:**
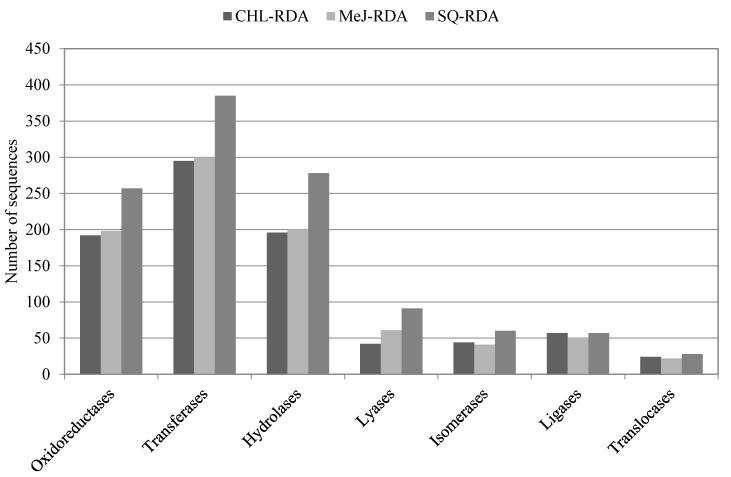
Enzyme classification of unigenes from *V. nigrum* based on Kyoto Encyclopedia of Genes and Genomes (KEGG) database.

Based on KEGG database, from 850 to 1156 unigenes representing three differential products matched with six classes of enzymes (Figure 6). Most sequences coded transferases (34%) and hydrolases (22%). Within the oxidoreductases, sequences corresponding to cytochrome P450 family accounted for 16, 15 and 18 in libraries developed after CHL, MeJ and SQ treatment, respectively.

### 3.4. Identification of Genes Involved in Terpenoid Backbone and Cholesterol Biosynthesis 

In plants, terpenoid backbone is synthesized from isopentenyl pyrophosphate (IPP) and dimethylallyl pyrophosphate (DMAPP) by cytosolic mevalonate (MVA) and plastidal 2-C-methyl-d-erythrirtol-4-phosphate (MEP) pathways [34]. 21 unigenes which encode all of the enzymes involved in the MVA route have been identified. In most cases these transcripts were overexpressed in subtraction libraries (FPKM values ranged from 4.82 to 401.47) (Table S2). Furthermore, in this pathway, the AACT enzyme was the best represented by 7 variants. Unlike the MVA pathway, two enzymes from the MEP route were missing. Transcripts encoding 4-diphosphocytidyl-2-C-methyl-D-erythritol kinase (CMK/IspE) and (E)-4-hydroxy-3-methylbut-2-enyl-diphosphate synthase (HDS/IspG) were not identified. Incomplete data of MEP pathway suggests that the MVA remains the main route in steroid skeletons production. In higher plants grown under normal physiological conditions, most of the carbon atoms in sterol molecules come from mevalonate [67]. In addition, experiments with incorporation of 14C-labeled cholesterol and acetate in the jervine and veratramine structures confirm that the main biosynthesis route of steroidal alkaloids in *Veratrum* plants begins from acetate converted to cholesterol via the mevalonic acid pathway [1].

Subsequent stages catalyzed by isopentenyl-diphosphate delta-isomerase (IPI), geranyl diphosphate synthase (DPS), farnesyl diphosphate synthase (FPS), squalene synthase (SQS) and squalene monooxygenase (SQE) lead to the 2,3-oxidosqualene chain formation. From one to eight variants of all of the abovementioned enzymes were identified in sequenced differential products with high FPKM values (from 5.25 to 2662.72).

Previous studies indicate that steroidal alkaloids are synthesized via cycloartenol and cholesterol [1]. Two possible cholesterol biosynthesis pathways from cycloartenol have been suggested [68]. These routes differ in the location of delta 24-sterol reductase (DWF1) in transformation. DWF1 can convert desmosterol to cholesterol in the last stage of biosynthesis [69] or reduce cycloartenol already in the initial stages of the pathway [70,71]. According to these studies, conversion of cycloartenol to cholesterol requires at least ten stages catalyzed by enzymes: sterol-4alpha-methyl oxidase (SMO), sterol-4alpha-carboxylate 3-dehydrogenase (HSD), 3-keto steroid reductase (3-KSR), cyclopropyl isomerase (CPI), sterol 14-demethylase (CYP51G1), delta14-sterol reductase (FK), cholestenol delta-isomerase (HYD1), delta(7)-sterol-C5(6)-desaturase (DWF7), 7-dehydrocholesterol reductase (DWF5) and delta 24-sterol reductase (DWF1). In RDA libraries, the HSD gene was represented by the highest number of unigenes and no transcript corresponding to HYD1 was identified (Table S2).

In conclusion, a total of 119 transcripts involved in steroidal backbone biosynthesis were found in the three differential RDA products, including 53 unigenes participating in transition from cycloartenol to cholesterol. The highest number of unigenes annotated to cholesterol synthesis (49 transcripts) was observed in the samples treated with squalene. A high range of FPKM values indicated that 93% of transcripts annotated to backbone biosynthesis route were overexpressed in variable level. 

### 3.5. Identification of the Genes in Later Stages of Jervine Biosynthesis

**Figure 7:**
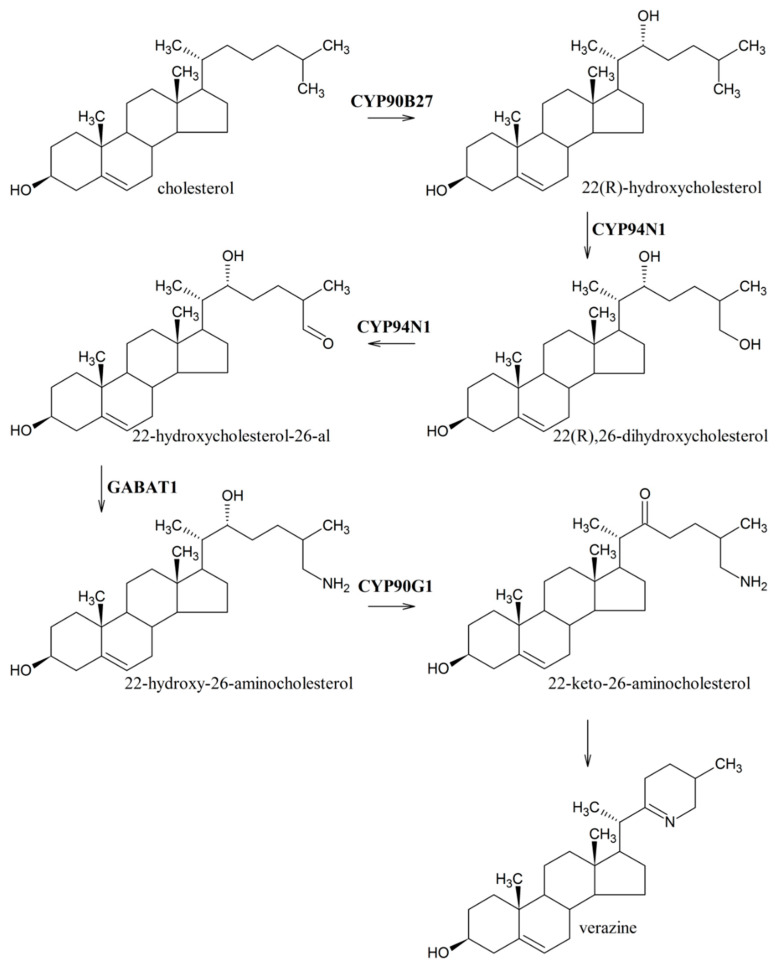
Verazine biosynthetic pathway in *Veratrum californicum* according to Augustin et al. [36].

At the molecular and biochemical level, information about genes involved in the downstream steps from cholesterol to jervine biosynthesis in *V. nigrum* plants is limited. Studies with ^14^C-labeled cyclopamine indicated its role as a possible precursor in jervine biosynthesis [72]. In *Veratrum californicum*, four enzymes catalyzing the first six steps of verazine biosynthesis (Figure 7) have been identified and functionally confirmed in Sf9 cells [36]. Verazine, which is the precursor of cyclopamine, can be biosynthesized from cholesterol by a series of hydroxylation/oxidation reactions at C-22 and C-26 and transamination at the C-26 position. The hydroxylation/oxidation of cholesterol is catalyzed by three specific cytochromes P450 (CYP90B27, CYP94N1 and CYP90G1), and introduction of amine group is mediated by γ-aminobutyrate transaminase (GABAT1). 

Alternative enzymes for the above chemical transitions have been reported. The steroid C-22 hydroxylation may also be catalyzed by CYP90B1 from *Arabidopsis thaliana* [73,74], CYP90B2 and CYP724B1 from *Oryza sativa* [75], CYP724B2 and CYP90B3 from *Lycopersicon esculentum* [76], CYP72A186 from *Solanum lycopersicum* [77] and CYP72A188 from *Solanum tuberosum* [78]. Activity of steroid C-26 hydroxylase/oxidase (CYP94N1), was previously reported for CYP734A1 from *Arabidopsis thaliana* [79], CYP734A7 from *Solanum lycopersicum* [80], CYP734A1 from *Oryza sativa* [81], CYP72A208 and CYP88B1 from *Solanum lycopersicum* [77]. The ability to convert 22-hydroxycholesterol-26-al to 22-hydroxy-26aminocholesterol was also confirmed for *S. lycopersicum* GABA transaminase isozyme 2 [36].

**Figure 8:**
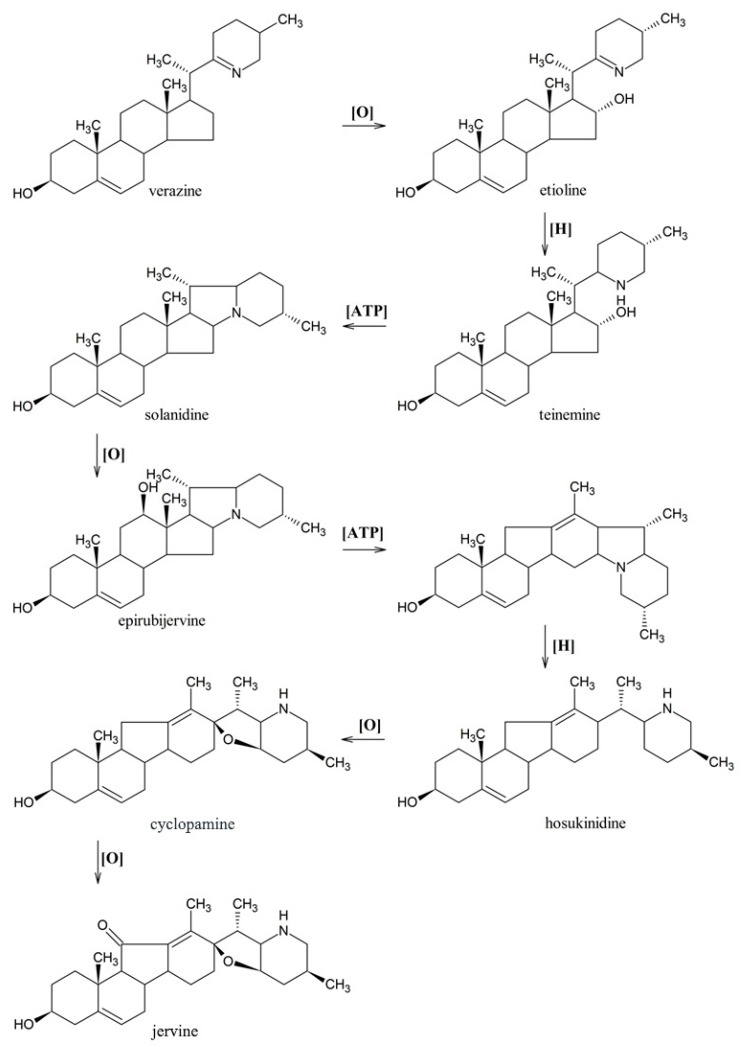
Proposed biosynthesis pathway of jervine from verazine [30].

Although the chemical mechanism of cyclopamine synthesis has been proposed [30], the genes involved in the downstream steps of transition from verazine to jervine remain largely unknown. The conversion of verazine to the *Veratrum* alkaloids featuring a C-nor-D-homosteroidal skeleton includes many stages. The E-ring of solanidine is formed after hydroxylation at C-16 position and the creation of a piperidine ring. Afterwards, stereo-unspecific oxidase hydroxylates at the C-12 position and 12-hydroxy product is rearranged to the C-nor-D homosteroidal skeleton by Wagner–Meerwein type reaction. Next, husokinidine is formed by reductive opening of the E ring (pyrrolidine ring) and is oxidatively cyclized to form the tetrahydrofuran ring of cyclopamine. Finally, hydroxylation and oxidation at C-11 cyclopamine leads to jervine alkaloid [1,30] (Figure 8). 

Previous reports indicate that multiple oxidations at the C-16, C-12 and C-11 positions may be catalyzed by enzymes from cytochrome P450 family and/or 2-oxoglutarate-dependent dioxygenase superfamily (2OGD). Generally, 2OGD enzymes are able to hydroxylate wide groups of phytochemicals, e.g., flavonoids, gibberellins and phytohormones. The 2-oxoglutarate-dependent dioxygenases from *Solanum tuberosum* (St16DOX) and *Solanum lycopersicum* (Sl16DOX) catalyze 16α-hydroxylation of hydroxycholesterols with high preference to the (22S)-22,26-dihydroxycholesterol as the best substrate [82,83]. In addition, several of CYP P450 were also proposed to catalyze C-16 oxidation reactions including CYP716A111 from *Aquilegia coerulea*, CYP716A141 from *Platycodon grandifloras*, CYP716Y1 from *Bupleurum falcatum* and CYP87D16 from *Maesa lanceolate* [84,85,86]. 

Moreover, cytochromes CYP87D18 from *Siraitia grosvenorii*, and CYP88D6 from *Glycyrrhiza glabra* were shown to catalyze C-11 hydroxylation, and CYP716A47 from *Panax ginseng* C-12 hydroxylation [87,88,89]. Additionally, unspecific monooxygenase (EC 1.14.14.1) from *Arabidopsis thaliana* may also catalyze oxidation reactions such as estrone to 16-hydroxyestrone, estradiol-17β to estriol and dehydroepiandrosterone to 16α-hydroxy dehydroepiandrosterone and can be involved in sterols oxidation at the C-16 in plants [46]. 

**Figure 9:**
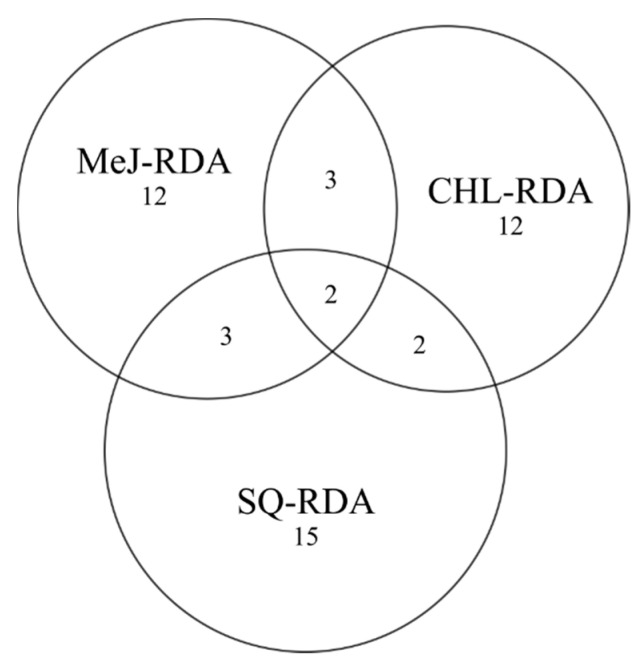
Venn diagram showing the distribution of unique and common transcripts coding cytochromes P450 in subtractive libraries (representational difference analysis - RDA) obtained after squalene (SQ), methyl jasmonate (MeJ) and cholesterol (CHL) treatment.

A total of 19, 20 and 22 CYP-encoding transcripts were found in CHL-RDA, MeJ-RDA and SQ-RDA libraries and were clustered into 49 unique sequences (Figure 9 and Table S3). These sequences were further grouped into 20 subfamilies (CYP51, CYP71, CYP73, CYP76, CYP81, CYP82, CYP83, CYP84, CYP86, CYP88, CYP93, CYP94, CYP97, CYP98, CYP701, CYP706, CYP710, CYP714, CYP734, CYP736). Eight transcripts with Pfam domain characteristic for P450 cytochromes were not classified within these subfamilies. In order to select the best candidate genes, these unigenes were blasted to 25 known reference sequences encoding enzymes responsible for hydroxylation/oxidation reactions at C-22 and C-26 and with other CYP genes with established C-11, C-12 and C-16 hydroxylation/oxidation activity (Table S4).

At the first stage, nine reference genes with C-22 hydroxylase activity were compared with RDA transcripts at protein level. The reference genes included two *Arabidopsis thaliana* splicing variants and sequences with established activity in verazine biosynthesis in closely related *V. californicum* (Table S4) [36]. The comparative analysis indicated the main candidate with C-22 hydroxylase activity. The candidate with C-22 hydroxylase activity refers to two contigs (1929 and 1930) specific to CHL-RDA libraries similar to potato PGA2 gene (Table 3 and Table S3).

The length of fragments generated by RDA procedure in the study was rather short (about 280 bp), therefore extended sequences of the corresponding genes (Tables S5, S7, S9 and S11) expressed in different organs of *V. nigrum* were explored [37]. The study has shown that contigs 1929 and 1930 correspond to contig_454 overexpressed (FPKM = 169.18) in *V. nigrum* roots (Table S5). Moreover, two sequences of *V. nigrum* homologous to CYP90B27 from *V. californicum* (stalk_contig_678, and root_contig_641) were found in stalk and root specific libraries (Table S5). RDA and organ-specific sequencing data indicate that root_contig_454 codes alternative enzyme with C-22 or C-16 hydroxylation activity in *V. nigrum*.

RDA procedure failed to indicate candidates involved in C-22 oxidation. Sequences of CHL-RDA_contig_263 and MeJ-RDA_contig_1967 were similar to reference gene CYP90G1 in *V. californicum* at low stringency, and showed homology to leaf_contig_45093 with putative C-16 hydroxylation activity (Table S3). However, sequences (root_contig_4383, stalk_contig_6773 and root_contig_510) coding variants of CYP90G1 in *V. nigrum* were identified in organs (Table S5). It seems that the RDA approach may not be effective in case of genes with multiple active variants showing high homology and may result in elimination of target fragments.

The RDA approach resulted in the selection of correct CHL-RDA_contig_3050 with C-26 hydroxylase activity that corresponds to leaf_contig_707 homologous to reference gene CYP94N1 in *V. californicum* [36]. The second candidate represented by MeJ-RDA_contig_1334 similar to tomato CYP734A7 genes was highly overexpressed in the analyzed RDA libraries (Table 3). Similarly, subtractive libraries retained their best candidate for γ-aminobutyrate transaminase (GABAT2) with established role in verazine biosynthesis [36]. Over 40 unigenes with Pfam domains characteristic for aminotransferases (PF00202, PF00155 and PF00266) were found within transcripts in subtractive libraries of *V. nigrum* (Table S6). Over 90% of these unigenes were overexpressed (FPKM value from 6.01 to 1604.27). Blast analyses clearly indicated MeJ-RDA_contig_1251 with the highest homology to the reference GABAT2 gene (Table 3). Five highly homologous to GABAT2 sequences were found in organs of *V. nigrum* (Table S7) including root_contig_544 matching MeJ-RDA_contig_1251. 

To reveal the candidate genes encoding enzymes participating in transition from verazine to cyclopamine, information from related species was used. Two C-11 hydroxylases/oxidases CYP87D18 from *Siraitia grosvenorii* [87] and CYP88D6 from *Glycyrrhiza glabra* [90] were used as references (Table S4). The results of RDA analysis indicate CHL-RDA_contig_648 (FPKM = 295) similar to CYP88D6 as putative candidate with C-11 activity (Table 3 and Table S3). Introduction of reference CYP716A47 from *Panax ginseng*, with steroid C-12 hydroxylase activity in ginsenoside biosynthesis pathway [91], was not successful in identifying homologous genes in *V. nigrum* (Table S4). 

Five reference genes coding enzymes with the C-16 oxidation activity in steroidal alkaloid biosynthesis were compared with RDA and organ transcripts (Tables S3–S5). The best candidate with C-16 hydroxylase activity was SQ-RDA_contig_3964 (FPKM = 41) homologous to *Arabidopsis thaliana* CYP86A2 corresponding to stalk_contig_5333 (Table 3). Additional genes similar to CYP86A2 at protein level were found in organ data. Alternatively, the C-16 oxidation might be catalyzed by substrate specific enzymes from the 2-oxoglutarate-dependent dioxygenase superfamily. Annotation of transcripts from subtractive libraries of *V. nigrum* resulted in 27 2OGD-encoding unigenes with characteristic Pfam domains (PF03171 and PF14226) corresponding to 21 contigs (Table S8). The comparative analysis to the 16DOX reference gene from *Solanum lycopersicum* [82] indicates MeJ-RDA_contig_2593 and MeJ-RDA_contig_81 as the best candidates with sterol C-16 oxidation activity, expressed in stalk and roots respectively (Table 3 and Tables S8 and S9).

In the last stages of jervine biosynthesis, two reactions were associated with the reduction of heterocycles containing one nitrogen atom, i.e., reduction of 2,3,4,5-tetrahydropyridine and then reduction of the pyrrolidine ring. Based on 98% amino acid identity to pyrroline-5-carboxylate reductase (EC 1.5.1.2) from *Medicago truncatula*, SQ-RDA_contig_2664 transcript (FPKM = 18.09) was selected as candidate able to catalyze etioline reduction (Table 3 and Table S10). This enzyme belongs to the oxidoreductases family, especially those which act on the CH-NH group of donors and are capable of using delta-1-piperideine-6-carboxylate as a substrate [92]. 

The reduction of C = N binding in plants might be also catalyzed by enzymes from short-chain dehydrogenases/reductases family (SDR), even in case of cyclic substrate [93]. The SDR enzyme family is one of the largest among all enzyme families and classifies approximately 46,000 members with differentiated activities, e.g., oxidoreductases, lyases and isomerases [94]. In the cDNA-RDA products, 21 unknown sequences containing the SDR domain were found (PF00106.20). Three unigenes (CHL-RDA_contig_2945, MeJ-RDA_contig_1516 and SQ-RDA_contig_1577) characterized with the highest FPKM values in the range between 3843.51 and 1967.9 (Table S10) may be selected as candidates involved in this process. 87 sequences with SDR domain were identified in organs of *V. nigrum* and 3 root specific contigs 13569, 13569 and 12724 matched 4 respective RDA products (Tables S10 and S11).

## 4. Conclusions

In this study, the next-generation sequencing (NGS) technology was applied to cDNA-RDA products obtained after cholesterol, methyl jasmonate and squalene treatment. cDNA-RDA procedure allowed the reduction of the number of overrepresented transcripts and increased the possibility of identifying low frequency, unique candidate genes involved in VSA biosynthesis, which were overexpressed by elicitation and precursor feeding. Here, a total of 12048 contigs were generated with a median length of 280 for three samples. As a result of the analysis, the majority of candidate genes involved in the unique VSA biosynthesis pathway have been identified. Transcripts encoding enzymes involved in C-22, C-26, C-11 and C-16 hydroxylation, transamination at C-26 and heterocycles reduction have been proposed. No transcript corresponding to C-22 oxidation and C-12 hydroxylation/oxidation was found. Furthermore, enzymes from the short-chain dehydrogenases/reductases family (SDR) associated with the reduction reactions in VSA biosynthesis pathway were identified. Further work is necessary to validate the role of the selected candidate genes in VSA biosynthesis.

## Figures and Tables

**Table 1:** Summary of sequence assembly data for cDNA-RDA products.

	CHL-RDA	MeJ-RDA	SQ-RDA
Total number of contigs	3547	3712	4789
Total bases in contigs (bp)	988,613	1,047,608	1,339,219
Min length of contigs (bp)	74	34	60
Max length of contigs (bp)	922	1003	886
Average length of contigs (bp)	279	282	280
GC content (%)	49.5	48.3	47.1
N75 (bp)	233	235	234
N50 (bp)	275	279	279
N25 (bp)	339	343	339
Number of contigs <500 bp	3480	3621	4708
Number of contigs ≥500 bp	67	91	81

**Table 2:** Total number of annotated *V. nigrum* unigenes in CHL-, MeJ- and SQ-RDA libraries.

Database	RDA Library		
	CHL	MeJ	SQ
NT	3175	3474	4379
NR	3005	3308	4188
KOG	1648	1721	2193
Pfam	1801	1858	2383
KEGG	1415	1438	1845
GO	2082	2647	3308
Total	3528	3662	4701

**Table 3:** Candidate genes involved in veratrum-type steroidal alkaloids (VSA) biosynthesis in *V. nigrum* plant.

Unigenes ID	Function	Reference Gene	Blastx E-Value
CHL-RDA_contig_1929	C-22 hydroxylation	PGA2 (*Solanum tuberosum*)	2.90 × 10^−12^
CHL-RDA_contig_1930	C-22 hydroxylation	PGA2 (*Solanum tuberosum*)	2.40 × 10^−12^
CHL-RDA_contig_3050	C-26 hydroxylation	CYP94N1v2 (*Veratrum californicum*)	9.27 × 10^−15^
MeJ-RDA_contig_1334	C-26 hydroxylation	CYP734A7 (*Solanum lycopersicum*)	3.00 × 10^−38^
MeJ-RDA_contig_1251	transamination	GABAT2 (*Veratrum californicum*)	2.51 × 10^−44^
CHL-RDA_contig_648	C-11 hydroxylation	CYP88D6 (*Glycyrrhiza glabra*)	2.59 × 10^−16^
SQ-RDA_contig_3964	C-16 hydroxylation	CYP86A2 (*Arabidopsis thaliana*)	1.05 × 10^−33^
MeJ-RDA_contig_2593	C-16 hydroxylation	16DOX (*Solanum lycopersicum*)	5.60 × 10^−26^
MeJ-RDA_contig_81	C-16 hydroxylation	16DOX (*Solanum lycopersicum*)	5.55 × 10^−12^
SQ-RDA_contig_2664	reduction of heterocycles	PYCR (*Medicago truncatula*)	2.00×10^−55^

## Supplementary Materials

The following are available online at https://www.mdpi.com/2075-1729/10/6/88/s1, Table S1: KEGG classification of the unigenes from *V. nigrum* after cDNA-RDA experiments; Table S2: Enzymes annotated to terpenoid backbone and sterols biosynthetic pathway with FPKM values. Table S3: Annotation of transcripts from subtractive libraries identified as CYP candidates; Table S4: Reference genes used for selection of candidates catalyzing stages in synthesis of jervine; Table S5: Annotation of transcripts from organs libraries identified as CYP candidates; Table S6: Annotation of transcripts from subtractive libraries identified as potential transaminases; Table S7: Annotation of transcripts from organs libraries identified as potential transaminases; Table S8: Annotation of transcripts from subtractive libraries identified as potential C16-hydroxylation candidates (from 2OGD enzymes family); Table S9: Annotation of transcripts from organs libraries identified as potential C16-hydroxylation candidates (from 2OGD enzymes family); Table S10. Annotation of transcripts associated with reduction of heterocycles from oxidoreductases family (unigene no 1) and from short-chain dehydrogenases/reductases family (SDR) (unigenes no 2–22) in all three differential products; Table S11: Characteristics of transcripts coding enzymes from short-chain dehydrogenases/reductases family (SDR) in all *V. nigrum* organs.

## References

[1] Chandler C.M., McDougal O.M. (2014). Medicinal history of North American *Veratrum*. Phytochem. Rev..

[2] Heretsch P., Giannis A. (2015). The *Veratrum* and Solanum alkaloids. Alkaloids Chem. Biol..

[3] Schaffner U., Kleijn D., Brown V., Müller-Schärer H. (2001). *Veratrum album* L. in montane grasslands: A model system for implementing biological control in land management practices of high biodiversity habitats. Biocontrol News Inf..

[4] Zomlefer W.B., Whitten W.M., Williams N.H., Judd W.S. (2003). An overview of Veratrum s.l. (Liliales: Melanthiaceae) and an infrageneric phylogeny based on ITS sequence data. Syst. Bot..

[5] Treier U.A., Müller-Schärer H. (2011). Differential effects of historical migration, glaciations and human impact on the genetic structure and diversity of the mountain pasture weed *Veratrum album* L.. J. Biogeogr..

[6] Szeliga M., Ciura J., Tyrka M. (2017). Genetic diversity of three European *Veratrum* species revealed by amplified fragment length polymorphism. Biodiv. Res. Conserv..

[7] Wu Z.Y., Raven P.H. (2000). Flora of China. Vol. 24 (Flagellariaceae through Marantaceae).

[8] Kaźmierczakowa R., Bloch-Orłowska J., Celka Z., Cwener A., Dajdok Z., Michalska-Hejduk D., Pawlikowski P., Szczęśniak E., Ziarnek K. (2016). Polish Red List of Pteridophytes and Flowering Plants.

[9] Barroso Mdo S. (2015). The hellebore, the plant beloved by the Greeks: The reasons behind a myth. Vesalius.

[10] Suladze T.S., Vachnadze V.Y., Tsakadze D.M., Gedevanishvili M.D., Tsutsunava L.E., Malazoniya N.A. (2006). Alkaloid accumulation dynamics in *Veratrum lobelianum* growing in Georgia and biological activity of Jervine. Chem. Nat. Compd..

[11] Cholakova M., Bratanov M., Christov V., Kostova N., Gantcheva M., Nikolova E. (2009). The veratrum alkaloid, veratroylzygadenine, suppresses contact dermatitis in mice. J. Med. Plants Res..

[12] Gaillard Y., Pepin G. (2001). LC-EI-MS determination of veratridine and cevadine in two fatal cases of *Veratrum album* poisoning. J. Anal. Toxicol..

[13] Wang Z.Z., Zhao W.J., Zhang X.S., Tian X.F., Wang Y.Z., Zhang F., Yuan J.C., Han G.Z., Xin K.X., Yao J.H. (2007). Protection of *Veratrum nigrum L. var. ussuriense Nakai alkaloids* against Ischemia-Reperfusion injury of the rat liver. World J. Gastroenterol..

[14] Wang L., Li W., Liu Y. (2008). Hypotensive effect and toxicology of total alkaloids and veratramine from roots and rhizomes of *Veratrum nigrum* L. in spontaneously hypertensive rats. Pharmazie.

[15] Li H.J., Jiang Y., Li P. (2006). Chemistry, bioactivity and geographical diversity of steroidal alkaloids from the Liliaceae family. Nat. Prod. Rep..

[16] Ivanova A., Serly J., Christov V., Stamboliyska B., Molnar J. (2011). Alkaloids derived from genus *Veratrum* and Peganum of Mongolian origin as multidrug resistance inhibitors of cancer cells. Fitoterapia.

[17] Dumlu F.A., Aydin T., Odabasoglu F., Berktas O.A., Kutlu Z., Erol H.E., Halici M.B., Cadirci E., Cakir A. (2019). Anti-Inflammatory and antioxidant properties of jervine, a sterodial alkaloid from rhizomes of *Veratrum album*. Phytomedicine.

[18] Li Q., Zhao Y.L., Long C.B., Zhu P.F., Liu Y.P., Luo X.D. (2019). Seven new Veratramine-Type alkaloids with potent analgesic effect from *Veratrum taliense*. J. Ethnopharmacol..

[19] Kang C., Han J.H., Oh J., Kulkarni R., Zhou W., Ferreira D., Jang T.S., Myung C.S., Na M. (2015). Steroidal alkaloids from *Veratrum nigrum* enhance glucose uptake in skeletal muscle cells. J. Nat. Prod..

[20] Incardona J.P., Eaton S. (2000). Cholesterol in signal transduction. Curr. Opin. Cell Biol..

[21] Chen J.K., Taipale J., Cooper M.K., Beachy P.A. (2002). Inhibition of hedgehog signaling by direct binding of cyclopamine to smoothened. Genes Dev..

[22] Shang Y., Du Q., Liu S., Staadler M., Wang S., Wang D. (2018). Antitumor activity of isosteroidal alkaloids from the plants in the genus *Veratrum* and *Fritillaria*. Curr. Protein Pept. Sci..

[23] Zhang X., Wang Y., Liang Q., Ma Z., Xiao C., Tan H., Gao Y. (2014). The correlation between chemical composition, as determined by UPLC-TOF-MS, and Acute toxicity of *Veratrum nigrum* L. and *Radix paeoniae alba*. Evid. Based Complement. Altern. Med..

[24] Cong Y., Jia W., Chen J., Song S., Wang J.H., Yang Y.H. (2007). Steroidal alkaloids from the roots and rhizomes of *Vertrum nigrum* L.. Helv. Chim. Acta.

[25] Christov V., Mikhova B., Ivanova A., Serly J., Molnar J., Selenge D., Solongo A., Kostova N., Gerelt-Od Y., Dimitrov D. (2010). Steroidal alkaloids of *Veratrum lobelianum* Bernh. and *Veratrum nigrum* L.. Z. Nat. C J. Biosci..

[26] Cong Y., Wang J.H., Wang R., Zeng Y.M., Liu C.D., Li X. (2008). A study on the chemical constituents of *Veratrum nigrum* L. processed by rice vinegar. J. Asian Nat. Prod. Res..

[27] Khanfar M.A., El Sayed K.A. (2013). The *Veratrum* alkaloids jervine, veratramine, and their analogues as prostate cancer migration and proliferation inhibitors: Biological evaluation and pharmacophore modeling. Med. Chem. Res..

[28] Tang J., Li H.L., Shen Y.H., Jin H.Z., Yan S.K., Liu R.H., Zhang W.D. (2008). Antitumor activity of extracts and compounds from the rhizomes of *Veratrum dahuricum*. Phytother. Res..

[29] Thayer S.P., Di Magliano M.P., Heiser P.W., Nielsen C.M., Roberts D.J., Lauwers G.Y., Qi Y.P., Gysin S., Del Castillo C.F., Yajnik V. (2003). Hedgehog is an early and late mediator of pancreatic cancer tumorigenesis. Nature.

[30] Heretsch P., Tzagkaroulaki L., Giannis A. (2010). Cyclopamine and Hedgehog signaling: Chemistry, biology, medical perspectives. Angew. Chem. Int. Ed. Engl..

[31] Masamune T., Takasugi M., Murai A., Kobayashi K. (1967). The synthesis of Jervine and related alkaloids. J. Am. Chem. Soc..

[32] Kutney J.P. (1977). Synthetic studies in the *Veratrum* Alkaloid Series. The Total Synthesis of Verarine, Veratramine, Jervine, Veratrobasine, and Verticine. Bioorg. Chem..

[33] Tang J., Li H.L., Shen Y.H., Jin H.Z., Yan S.K., Liu R.H., Zhang W.D. (2008). Simultaneous determination of six steroidal alkaloids of *Veratrum* dahuricum by HPLC–ELSD and HPLC–MSn. Chromatographia.

[34] Suzuki M., Muranaka T. (2007). Molecular genetics of plant sterol backbone synthesis. Lipids.

[35] Sun C., Sun Y., Song J., Li C., Li X., Zhang X., Li Y., Hu S., Luo H., Zhu Y. (2011). Discovery of genes related to steroidal alkaloid biosynthesis in *Fritillaria cirrhosa* by generating and mining a dataset of expressed sequence tags (ESTs). J. Med. Plant Res..

[36] Augustin M.M., Ruzicka D.R., Shukla A.K., Augustin J.M., Starks C.M., O’Neil-Johnson M., McKain M.R., Evans B.S., Barrett M.D., Smithson A. (2015). Elucidating steroid alkaloid biosynthesis in *Veratrum californicum*: Production of verazine in Sf9 cells. Plant J..

[37] Szeliga M., Ciura J., Grzesik M., Tyrka M. (2019). Identification of candidate genes involved in steroidal alkaloids biosynthesis in Organ-Specific transcriptomes of *Veratrum nigrum* L.. Gene.

[38] Ma R., Yu Z., Cai Q., Li H., Dong Y., Oksman-Caldentey K.M., Rischer H. (2020). Agrobacterium-Mediated genetic transformation of the medicinal plant *Veratrum dahuricum*. Plants.

[39] Engler C., Kandzia R., Marillonnet S.A. (2008). One pot, one step, precision cloning method with high throughput capability. PLoS ONE.

[40] Facchini P.J., Bohlmann J., Covello P.S., De Luca V., Mahadevan R., Page J.E., Ro D.K., Sensen C.W., Storms R., Martin V.J. (2012). Synthetic biosystems for the production of High-Value plant metabolites. Trends Biotechnol..

[41] Singh A., Menéndez-Perdomo I.M., Facchini P.J. (2019). Benzylisoquinoline alkaloid biosynthesis in opium poppy: An update. Phytochem. Rev..

[42] Hubank M., Schatz D.G. (1994). Identifying differences in mRNA expression by representational difference analysis of cDNA. Nucleic Acids Res..

[43] Sasheva P., Grossniklaus U. (2017). Differentially methylated Region-Representational difference analysis (DMR-RDA): A powerful method to identify DMRs in uncharacterized genomes. Methods Mol. Biol..

[44] Haslinger C., Sommergruber W., Voss T., Schreiber M., Hayat M.A. (2006). Identification of Tumor-Specific genes. Handbook of Immunohistochemistry and In Situ Hybridization of Human Carcinomas.

[45] Hubank M., Schatz D.G. (1999). cDNA representational difference analysis: A sensitive and flexible method for identification of differentially expressed genes. Methods Enzymol..

[46] Ciura J., Szeliga M., Grzesik M., Tyrka M. (2017). Next-Generation sequencing of representational difference analysis products for identification of genes involved in diosgenin biosynthesis in fenugreek (*Trigonella Foenum-Graecum*). Planta.

[47] Hagel J.M., Morris J.S., Lee E.J., Desgagné-Penix I., Bross C.D., Chang L., Chen X., Farrow S.C., Zhang Y., Soh J. (2015). Transcriptome analysis of 20 taxonomically related benzylisoquinoline Alkaloid-Producing plants. BMC Plant Biol..

[48] Murashige T., Skoog F. (1962). A revised medium for rapid growth and bioassays with tobacco tissue cultures. Physiol. Plant..

[49] Huang Y., Niu B., Gao Y., Fu L., Li W. (2010). CD-HIT Suite: A web server for clustering and comparing biological sequences. Bioinformatics.

[50] Wu S., Zhu Z., Fu L., Niu B., Li W. (2011). WebMGA: A customizable web server for fast metagenomic sequence analysis. BMC Genom..

[51] Moriya Y., Itoh M., Okuda S., Yoshizawa A., Kanehisa M. (2007). KAAS: An automatic genome annotation and pathway reconstruction server. Nucleic Acids Res..

[52] Conesa A., Gotz S., Garcia-Gomez J.M., Terol J., Talon M., Robles M. (2005). Blast2GO: A universal tool for annotation, visualization and analysis in functional genomics research. Bioinformatics.

[53] Ye J., Fang L., Zheng H., Zhang Y., Chen J., Zhang Z., Wang J., Li S., Li R., Bolund L. (2006). WEGO: A web tool for plotting GO annotations. Nucleic Acids Res..

[54] Tian F., Yang D.C., Meng Y.Q., Jin J.P., Gao G. (2020). PlantRegMap: Charting functional regulatory maps in plants. Nucleic Acids Res..

[55] Thagun C., Imanishi S., Kudo T., Nakabayashi R., Ohyama K., Mori T., Kawamoto K., Nakamura Y., Katayama M., Nonaka S. (2016). Jasmonate-Responsive ERF transcription factors regulate steroidal glycoalkaloid biosynthesis in tomato. Plant Cell Physiol..

[56] Lee M.H., Cheng J.J., Lin C.Y., Chen Y.J., Lu M.K. (2007). Precursor-Feeding strategy for the production of solanine, solanidine and solasodine by a cell culture of *Solanum lyratum*. Process Biochem..

[57] Llorca C.M., Potschin M., Zentgraf U. (2014). bZIPs and WRKYs: Two large transcription factor families executing two different functional strategies. Front. Plant Sci..

[58] Suttipanta N., Pattanaik S., Kulshrestha M., Patra B., Singh S.K., Yuan L. (2011). The transcription factor CrWRKY1 positively regulates the terpenoid indole alkaloid biosynthesis in *Catharanthus roseus*. Plant Physiol..

[59] Xu Y.H., Wang J.W., Wang S., Wang J.Y., Chen X.Y. (2004). Characterization of GaWRKY1, a cotton transcription factor that regulates the sesquiterpene synthase gene (+)-δ-cadinene synthase-A. Plant Physiol..

[60] Li S., Zhang P., Zhang M., Fu C., Yu L. (2013). Functional analysis of a WRKY transcription factor involved in transcriptional activation of the DBAT gene in *Taxus chinensis*. Plant Biol. (Stuttg.).

[61] Ji Y., Xiao J., Shen Y., Ma D., Li Z., Pu G., Li X., Huang L., Liu B., Ye H. (2014). Cloning and characterization of AabHLH1, a bHLH transcription factor that positively regulates artemisinin biosynthesis in *Artemisia annua*. Plant Cell Physiol..

[62] Spyropoulou E.A., Haring M.A., Schuurink R.C. (2014). RNA Sequencing on *Solanum lycopersicum* trichomes identifies transcription factors that activate terpene synthase promoters. BMC Genom..

[63] Mertens J., Pollier J., Vanden Bossche R., Lopez-Vidriero I., Franco-Zorrilla J.M., Goossens A. (2016). The bHLH transcription factors TSAR1 and TSAR2 regulate triterpene saponin biosynthesis in *Medicago truncatula*. Plant Physiol..

[64] Reddy V.A., Wang Q., Dhar N., Kumar N., Venkatesh P.N., Rajan C., Panicker D., Sridhar V., Mao H.Z., Sarojam R. (2017). Spearmint R2R3-MYB transcription factor MsMYB negatively regulates monoterpene production and suppresses the expression of geranyl diphosphate synthase large subunit (*MsGPPS.LSU*). Plant Biotechnol. J..

[65] Cárdenas P.D., Sonawane P.D., Pollier J., Vanden Bossche R., Dewangan V., Weithorn E., Tal L., Meir S., Rogachev I., Malitsky S. (2016). GAME9 regulates the biosynthesis of steroidal alkaloids and upstream isoprenoids in the plant mevalonate pathway. Nat. Commun..

[66] Afrin S., Huang J.J., Luo Z.Y. (2015). JA-Mediated transcriptional regulation of secondary metabolismin medicinal plants. Sci. Bull..

[67] Schaller H. (2004). New aspects of sterol biosynthesis in growth and development of higher plants. Plant Physiol. Biochem..

[68] Sawai S., Ohyama K., Yasumoto S., Seki H., Sakuma T., Yamamoto T., Takebayashi Y., Kojima M., Sakakibara H., Aoki T. (2014). Sterol side chain reductase 2 is a key enzyme in the biosynthesis of cholesterol, the common precursor of toxic steroidal glycoalkaloids in potato. Plant Cell.

[69] Diener A.C., Li H., Zhou W., Whoriskey W.J., Nes W.D., Fink G.R. (2000). Sterol methyltransferase 1 controls the level of cholesterol in plants. Plant Cell.

[70] Nahar N., Westerberg E., Arif U., Huchelmann A., Olarte Guasca A., Beste L., Dalman K., Dutta P.C., Jonsson L., Sitbon F. (2017). Transcript profiling of two potato cultivars during Glycoalkaloid-Inducing treatments shows differential expression of genes in sterol and glycoalkaloid metabolism. Sci. Rep..

[71] Moreau R.A., Nyström L., Whitaker B.D., Winkler-Moser J.K., Baer D.J., Gebauer S.K., Hicks K.B. (2018). Phytosterols and their derivatives: Structural diversity, distribution, metabolism, analysis, and Health-Promoting uses. Prog. Lipid Res..

[72] Kaneko K., Mitsuhashi H., Hirayama K., Ohmori S. (1970). 11-Deoxojervine as a precursor for jervine biosynthesis in *Veratrum grandiflorum*. Phytochemistry.

[73] Choe S., Dilkes B.P., Fujioka S., Takatsuto S., Sakurai A., Feldmann K.A. (1998). The DWF4 gene of arabidopsis encodes a cytochrome P450 that mediates multiple 22α-Hydroxylation steps in brassinosteroid biosynthesis. Plant Cell.

[74] Fujita S., Ohnishi T., Watanabe B., Yokota T., Takatsuto S., Fujioka S., Yoshida S., Sakata K., Mizutani M. (2006). Arabidopsis CYP90B1 catalyses the early C-22 hydroxylation of C27, C28 and C29 sterols. Plant J..

[75] Sakamoto T., Morinaka Y., Ohnishi T., Sunohara H., Fujioka S., Ueguchi-Tanaka M., Mizutani M., Sakata K., Takatsuto S., Yoshida S. (2005). Erect leaves caused by brassinosteroid deficiency increase biomass production and grain yield in rice. Nat. Biotechnol..

[76] Ohnishi T., Watanabe B., Sakata K., Mizutani M. (2006). CYP724B2 and CYP90B3 function in the early C-22 hydroxylation steps of brassinosteroid biosynthetic pathway in tomato. Biosci. Biotechnol. Biochem..

[77] Itkin M., Heinig U., Tzfadia O., Bhide A.J., Shinde B., Cardenas P.D., Bocobza S.E., Unger T., Malitsky S., Finkers R. (2013). Biosynthesis of antinutritional alkaloids in solanaceous crops is mediated by clustered genes. Science.

[78] Umemoto N., Nakayasu M., Ohyama K., Yotsu-Yamashita M., Mizutani M., Seki H., Saito K., Muranaka T. (2016). Two cytochrome P450 Monooxygenases catalyze early hydroxylation steps in the potato steroid glycoalkaloid biosynthetic pathway. Plant Physiol..

[79] Thornton L.E., Rupasinghe S.G., Peng H., Schuler M.A., Neff M.M. (2010). Arabidopsis CYP72C1 is an atypical cytochrome P450 that inactivates brassinosteroids. Plant Mol. Biol..

[80] Ohnishi T., Nomura T., Watanabe B., Ohta D., Yokota T., Miyagawa H., Sakata K., Mizutani M. (2006). Tomato cytochrome P450 CYP734A7 functions in brassinosteroid catabolism. Phytochemistry.

[81] Sakamoto T., Kawabe A., Tokida-Segawa A., Shimizu B., Takatsuto S., Shimada Y., Fujioka S., Mizutani M. (2011). Rice CYP734As function as multisubstrate and multifunctional enzymes in brassinosteroid catabolism. Plant J..

[82] Nakayasu M., Umemoto N., Ohyama K., Fujimoto Y., Lee H.J., Watanabe B., Muranaka T., Saito K., Sugimoto Y., Mizutani M. (2017). A dioxygenase catalyzes steroid 16α-Hydroxylation in steroidal glycoalkaloid biosynthesis. Plant Physiol..

[83] Kawai Y., Ono E., Mizutani M. (2014). Evolution and diversity of the 2-oxoglutaratedependent dioxygenase superfamily in plants. Plant J..

[84] Miettinen K., Pollier J., Buyst D., Arendt P., Csuk R., Sommerwerk S., Moses T., Mertens J., Sonawane P.D., Pauwels L. (2017). The ancient CYP716 family is a major contributor to the diversification of eudicot triterpenoid biosynthesis. Nat. Commun..

[85] Tamura K., Teranishi Y., Ueda S., Suzuki H., Kawano N., Yoshimatsu K., Saito K., Kawahara N., Muranaka T., Seki H. (2017). Cytochrome P450 monooxygenase CYP716A141 is a Unique b-Amyrin C-16b oxidase involved in triterpenoid saponin biosynthesis in *Platycodon grandiflorus*. Plant Cell Physiol..

[86] Moses T., Pollier J., Almagro L., Buyst D., Van Montagu M., Pedreño M.A., Martins J.C., Thevelein J.M., Goossens A. (2014). Combinatorial biosynthesis of sapogenins and saponins in Saccharomyces cerevisiae using a C-16α hydroxylase from *Bupleurum falcatum*. Proc. Natl. Acad. Sci. USA.

[87] Zhang J., Dai L., Yang J., Liu C., Men Y., Zeng Y., Cai Y., Zhu Y., Sun Y. (2016). Oxidation of cucurbitadienol catalyzed by cyp87d18 in the biosynthesis of mogrosides from *Siraitia grosvenorii*. Plant Cell Physiol..

[88] Ghosh S. (2017). Triterpene structural diversification by plant cytochrome P450 enzymes. Front. Plant Sci..

[89] Hamberger B., Bak S. (2013). Plant P450s as versatile drivers for evolution of Species-Specific chemical diversity. Philos. Trans. R. Soc. B.

[90] Seki H., Ohyama K., Sawai S., Mizutani M., Ohnishi T., Sudo H., Akashi T., Aoki T., Saito K., Muranaka T. (2008). Licorice Beta-Amyrin 11-Oxidase, a cytochrome P450 with a key role in the biosynthesis of the triterpene sweetener glycyrrhizin. Proc. Natl. Acad. Sci. USA.

[91] Han J.Y., Kim H.J., Kwon Y.S., Choi Y.E. (2011). The Cyt P450 enzyme CYP716A47 catalyzes the formation of protopanaxadiol from Dammarenediol-II during ginsenoside biosynthesis in *Panax ginseng*. Plant Cell Physiol..

[92] Jeske L., Placzek S., Schomburg I., Chang A., Schomburg D. (2019). BRENDA in 2019: A European ELIXIR core data resource. Nucleic Acids Res..

[93] Roth S., Kilgore M.B., Kutchan T.M., Müller M. (2018). Exploiting the catalytic diversity of Short-Chain dehydrogenases/reductases: Versatile enzymes from plants with extended imine substrate scope. ChemBioChem.

[94] Persson B., Kallberg Y., Bray J.E., Bruford E., Dellaporta S.L., Favia A.D., Duarte R.G., Jörnvall H., Kavanagh K.L., Kedishvili N. (2009). The SDR (Short-Chain dehydrogenase/reductase and related enzymes) nomenclature initiative. Chem. Biol. Interact..

